# Hepatic monoamine oxidase B is involved in endogenous geranylgeranoic acid synthesis in mammalian liver cells^[S]^

**DOI:** 10.1194/jlr.RA119000610

**Published:** 2020-02-24

**Authors:** Yuki Tabata, Yoshihiro Shidoji

**Affiliations:** Molecular and Cellular Biology, Graduate School of Human Health Science, University of Nagasaki, Nagayo, Nagasaki 851-2195, Japan

**Keywords:** cancer, cancer prevention, isoprenoids, mass spectrometry, mevalonate pathway, retinoids, diterpenoid

## Abstract

Geranylgeranoic acid (GGA) originally was identified in some animals and has been developed as an agent for preventing second primary hepatoma. We previously have also identified GGA as an acyclic diterpenoid in some medicinal herbs. Recently, we reported that in human hepatoma-derived HuH-7 cells, GGA is metabolically labeled from ^13^C-mevalonate. Several cell-free experiments have demonstrated that GGA is synthesized through geranylgeranial by oxygen-dependent oxidation of geranylgeraniol (GGOH), but the exact biochemical events giving rise to GGA in hepatoma cells remain unclear. Monoamine oxidase B (MOAB) has been suggested to be involved in GGOH oxidation. Here, using two human hepatoma cell lines, we investigated whether MAOB contributes to GGA biosynthesis. Using either HuH-7 cell lysates or recombinant human MAOB, we found that: *1*) the MAO inhibitor tranylcypromine dose-dependently downregulates endogenous GGA levels in HuH-7 cells; and *2*) siRNA-mediated *MAOB* silencing reduces intracellular GGA levels in HuH-7 and Hep3B cells. Unexpectedly, however, CRISPR/Cas9-generated *MAOB*-KO human hepatoma Hep3B cells had GGA levels similar to those in *MAOB*-WT cells. A sensitivity of GGA levels to siRNA-mediated MAOB downregulation was recovered when the *MAOB*-KO cells were transfected with a *MAOB*-expression plasmid, suggesting that MAOB is the enzyme primarily responsible for GGOH oxidation and that some other latent metabolic pathways may maintain endogenous GGA levels in the *MAOB*-KO hepatoma cells. Along with the previous findings, these results provide critical insights into the biological roles of human MAOB and provide evidence that hepatic MAOB is involved in endogenous GGA biosynthesis via GGOH oxidation.

Geranylgeranoic acid (all-*trans* 3,7,11,15-tetramethyl-2,6,10,14-hexadecatetraenoic acid or GGA), first recognized as a mevalonate (MVA)-derived metabolite in cell-free homogenates of the bovine retina in 1983 (1) and then in a parasitic worm in 1993 (2), is a compound consisting of 4-isoprene units linked in a tail-to-head manner. GGA and its didehydro derivative were shown to be potent ligands for nuclear retinoid receptors (3), so these isoprenoid compounds have been developed as preventive agents against second primary hepatoma (4, 5). In the past, we reported that GGA is a natural compound present in some medicinal herbs (6). Recently, we found that GGA is not only present in plant tissues but is also endogenously present in various organs of male Wistar rats. Its biosynthesis from MVA via farnesyl diphosphate (FPP) and geranylgeranyl diphosphate (GGPP) is also confirmed in human hepatoma-derived cells (7). A previous study reported that GGPP added in rat liver homogenates is converted to geranylgeraniol (GGOH) by geranylgeranyl pyrophosphatase (GGPPase), which is most active at physiologic pH and highly specific for GGPP (8). GGOH produced by GGPPase had been thought to be oxidized to geranylgeranial (GGal) by cytosolic alcohol dehydrogenase (ADH) in the presence of NAD^+^ (9), and GGal had been supposed to be further oxidized to GGA by nonspecific aldehyde dehydrogenase (9). Indeed, we have confirmed that the enzymatic conversion from GGal to GGA is highly dependent on exogenous NAD^+^ in rat liver homogenates (10) and human hepatoma-derived HuH-7 cell lysates (11). However, we found that a putative enzyme in either rat liver or HuH-7 cells involved in the oxidation of GGOH to GGal did not require any exogenous NAD^+^ in the cell-free system (10, 11). The GGOH oxidation activity was highest in the mitochondrial fraction prepared from rat liver homogenates (10).

Taking account that the mitochondrial enzyme was sensitive to tranylcypromine (TCP), an inhibitor against monoamine oxidases (MAOs) (11), we have reasonably speculated that a certain member of the MAO family is involved in GGOH oxidation to GGal in the process of GGA biosynthesis (10, 11). We so far have the following three lines of evidence for MAOB as a GGOH-oxidizing enzyme: *1*) HuH-7 cell lysate or rat liver homogenate enzyme does not require the exogenous NAD^+^ to produce GGal; *2*) molecular oxygen solubilized in the reaction mixture is consumed upon addition of GGOH into HuH-7 cell lysates as an enzyme source; and *3*) the recombinant human MAOB protein actively oxidizes GGOH to GGal (11).

MAOB, a flavin enzyme located to the outer mitochondrial membrane, generally degrades phenylethylamine and dopamine in the central nervous system (12). When these substrates are oxidized by MAOB, molecular oxygen is consumed and reactive oxygen species, such as hydrogen peroxide, are generated. Therefore, an increase in the expression level of MAOB in the brain is expected to damage the nervous system due to the reactive oxygen species produced (13). In fact, the expression of MAOB is increased in Alzheimer’s disease and Parkinson’s disease (14, 15), and MAOB inhibitors have been investigated as treatments for these diseases (16, 17). In contrast to the diverse MAOB studies in the nervous system, the physiological role of MAOB in the liver is not clearly defined, despite the tissue levels of *MAOB* mRNA expression not only being higher than those in the central nervous system but also being highest among all human organs (18). At present, the liver MAOB enzyme is considered to contribute to the decomposition of xenobiotics because the liver is a major organ that contributes to drug metabolism, and MAOB shows relatively broad substrate specificity for aromatic amines (19). In this context, the physiological substrate of liver MAOB has not yet been clearly elucidated. Therefore, if we can show that GGOH is an endogenous substrate of hepatic MAOB, as mentioned earlier, we will add a new perspective on the physiological role of hepatic MAOB.

By using an MAO inhibitor and siRNAs to inhibit and downregulate the cellular MAOB enzyme activity, here, we demonstrate that hepatic MAOB is involved in the maintenance of the intracellular GGA level in human hepatoma-derived cells. To ensure that MAOB is involved in GGA biosynthesis more reliably, we performed KO of the *MAOB* gene using the CRISPR/Cas9 plasmids in human hepatoma cells, but, unexpectedly, the intracellular GGA content of *MAOB*- KO cells was almost the same as that of the WT cells. However, when the *MAOB*-KO cells were back-transfected with *MAOB* expression plasmid, *MAOB* siRNA-mediated downregulation of the endogenous GGA level was recovered. In other words, when MAOB is expressed normally in human hepatocytes, the intracellular level of GGA is dependent on MAOB activity. The possibility that enzymes other than MAOB in *MAOB*-KO cells are involved in maintaining the intracellular GGA content is also described.

## MATERIALS AND METHODS

### Chemicals

All-*trans* GGA and 2,3-dihydroGGA were prepared by Kuraray Co. (Okayama, Japan) and Kowa Pharmaceutical (Tokyo, Japan). GGOH was provided by Eisai Foods (99% pure; Tokyo, Japan). Acetonitrile (LC/MS grade), ethanol, farnesol (FOH), geraniol (GOH), hygromycin, Dulbecco’s PBS, without calcium chloride and magnesium chloride and suitable for cell culture [PBS (−)], and TCP were all purchased from Sigma-Aldrich (St. Louis, MO). Citral, methanol, and DMEM (high glucose) were from Wako Pure Chemical Industries (Osaka, Japan). Chloroform was obtained from Kanto Chemical Co. (Tokyo, Japan). FBS was bought from HyClone, Thermo Fisher (Tokyo, Japan). Zaragozic acid A (ZAA; an inhibitor of squalene synthase) was provided from Merck (Darmstadt, Germany). All chemicals other than those stated above were of reagent grade.

### Cell culture

Human hepatoma-derived HuH-7 and Hep3B cells were maintained with DMEM containing 5% FBS at 37°C in a humidified atmosphere of 5% CO_2_.

### Treatment of HuH-7 cells with MAO inhibitor

HuH-7 cells (5 × 10^5^ cells/dish, in a 10 cm diameter dish) were inoculated and cultured in DMEM containing 5% FBS for 24 h; thereafter, the medium was replaced with FBS-free DMEM 1 day before TCP treatment. After 24 h treatment of the cells with different concentrations (0–100 μM) of TCP or with 100 μM of TCP + 25 μM of GGOH, the cells were harvested using a plastic cell lifter (Nunc, Roskilde, Denmark) and the cellular GGA was quantitatively measured using LC/MS/MS as described below. The IC_50_ of TCP on the endogenous GGA levels was calculated using GraphPad Prism version 7 for Windows (GraphPad Software, La Jolla, CA).

### Transfection with siRNA

Ready-made siRNAs for the *MAOB*, *MAOA*, *ADH1A*, and prenylcysteine oxidase 1 (*PCYOX1*) genes were purchased from Santa Cruz Biotechnology (Santa Cruz, CA). The siRNAs included three pairs of about 20 nt siRNAs designed to knock down the expression of each specific gene (see supplemental Table S1). For transfection, HuH-7 cells or Hep3B cells were inoculated on 30 mm dishes at a density of 4 × 10^4^ cells/dish. On the next day, 80 pmol of each siRNA were transfected using Lipofectamine® 2000 (Thermo Fisher Scientific). After a 72 h incubation, total RNA was prepared from cells to measure the cellular mRNA levels of each gene. Cells were incubated for an additional 48 h (120 h in total after transfection) and used for LC/MS/MS quantification of intracellular GGA.

In addition, after HuH-7 cells were incubated with *MAOB* siRNA for 96 h, the cells were incubated for another 24 h in the 25 μM GGOH or 15 μM ZAA.

### KO of the *MAOB* gene in hepatoma cells by using the CRISPR/Cas9 system

HuH-7 or Hep3B cells (3 × 10^4^ cells/dish, in a 30 mm-diameter dish) were transfected with 1 μg of *MAOB* CRISPR/Cas9 KO plasmids and 1 μg of HDR plasmids (Santa Cruz Biotechnology). After an approximately 2 week screening with 2 μg/ml puromycin, Hep3B, but no HuH-7 cell clones, were established by confirming RFP fluorescence on a laser-scanning confocal fluorescence microscope (LSM-700; Carl Zeiss, Berlin, Germany; see supplemental Fig. S1) and a lack of both *MAOB* mRNA expression using the QRTPCR method and MAOB protein expression using the Western blotting method were confirmed in these Hep3B/*MAOB*-KO cells.

### Back-transfection of the *MAOB* gene into Hep3B/*MAOB*-KO cells

Hep3B/*MAOB*-KO cells (3 × 10^4^ cells/dish, in a 30 mm-diameter dish) were transfected with 1 μg of human *MAOB* gene ORF (open reading frame) cDNA clone expression plasmid (Sino Biological, Beijing, China). After an approximately 2 week selection with 200 μg/ml hygromycin, re-expression of both *MAOB* mRNA using the QRTPCR method and MAOB protein using the Western blotting method were confirmed in Hep3B/*MAOB*-KO/TG cells.

### QRTPCR

Total RNA was prepared from each cell culture using the Fastgene™ RNA Basic kit (Nippon Genetics, Tokyo, Japan). For cDNA synthesis, Fastgene™ Scriptase II (Nippon Genetics) was used according to the manufacturer’s instructions. Real-time PCR was performed using LightCycler FastStart DNA Master^PLUS^ SYBR Green I (Roche Diagnostics, Tokyo, Japan) on a LightCycler 96 (Roche). Gene expression levels were analyzed using the 2^−ΔΔCt^ method. Primer sequences and real-time PCR settings used in this study are presented in supplemental Tables S2–S4.

### Immunoblotting

Proteins were prepared from the cells with RIPA lysis buffer (Merck Millipore, Tokyo, Japan) containing protease inhibitors (cOmplete™ Protease Inhibitor Cocktail; Roche Diagnostic) and the solubilized proteins were quantified by Bradford assay (Bio-Rad, Hercules, CA). Equal amounts (10 μg) of protein per sample were separated by Mini-PROTEAN TGX precast gels (Bio-Rad) and transferred onto polyvinylidene fluoride membranes. Horseradish peroxidase-labeled secondary antibodies (GE Healthcare, Tokyo, Japan) were detected with an ECL plus Western blotting detection system (GE Healthcare) or a SuperSignal™ West Femto maximum sensitivity substrate (Thermo Fisher Scientific) using an ImageQuant LAS 4000 (GE Healthcare).

### Chemical synthesis and purification of GGal, farnesal, and geranial

GGOH (29 mg), FOH (22 mg), or GOH (15 mg) were each treated with 300 mg of active MnO_2_ (Sigma-Aldrich) in 3 ml of chloroform at 40°C for 48 h. The reaction mixture was centrifuged at 300 *g* for 10 min to remove pellets. The chloroform extracts containing each aldehyde were dried under a nitrogen gas stream and dissolved in ethanol and purified by chromatography with a LiChroprep RP-18 column (2.5 × 31 cm; Merck). The mobile phase was methanol at a flow rate of 2.0 ml/min. Each purified aldehyde was dried under nitrogen gas and dissolved in ethanol. Each synthesized aldehyde was monitored by its characteristic UV absorption spectrum at 245 nm with citral as a standard and further use for LC/MS/MS calibration as a standard for each aldehyde.

### Enzyme assays

Aliquots of 3–100 μM GGOH, FOH, or GOH were incubated at 37°C with the recombinant human MAOB protein (0.15 μg, >85% pure; Active Motif, Carlsbad, CA) in a final volume of 100 μl of water. The reaction was then stopped by chilling on ice, and the reaction mixture was diluted with 9 vol of ethanol. The resultant ethanolic extract was filtered through a Cosmonice Filter S cartridge (0.45 μm) prior to the analyses of GGal, farnesal (Fal), and geranial (Gal) by LC/MS/MS. The *K_m_* for each substrate was calculated using GraphPad Prism 7.

### Lipid extraction and quantitative measurement of the cellular GGA

HuH-7 or Hep3B cells and the conditioned medium were separately collected in each tube by centrifugation (200 *g*, 8 min). To extract the total cellular lipids, the cell pellets were added to chloroform/methanol (2:1 v/v; 20-fold volumes over cell volume) and sonicated on ice (three times; 30 s each). After standing overnight at room temperature and being centrifuged (200 *g*, 8 min), the supernatant was transferred to a screw-capped glass tube and evaporated to dryness under a nitrogen stream. The residues were dissolved with 100 μl of ethanol and filtered through a Cosmonice Filter S cartridge (0.45 μm) just prior to LC/MS/MS analysis.

### LC/MS/MS analysis

GGA was detected by the procedures described in our previous study (7). The chromatographic run to measure GGOH and GGal was operated by linear gradients between solution A (milli-Q water containing 0.1% formic acid) and solution B (acetonitrile containing 0.1% formic acid). The elution was conducted at a constant flow rate of 0.30 ml/min as follows: 0–12 min, isocratic 74% B; 2–13 min, a linear ascending gradient from 74% B to 100% B; 13–18 min, 100% B; 18–19 min, a linear descending gradient from 100% B to 74% B; 19–22 min, 74% B. The specific combination of the molecular ion and fragment ion, cone voltage, and collision cell energy for each compound are listed in supplemental Table S5. Other conditions were the same as the previous procedure (7).

### Statistical analysis

Statistical comparisons were performed using a *t*-test or ANOVA with post hoc Scheffe test where appropriate. All data, unless specified, are presented as mean ± SE, with a statistically significant difference defined as *P* < 0.05.

## RESULTS

### Inhibition of the biosynthesis of cellular GGA with TCP

**Fig. 1.**
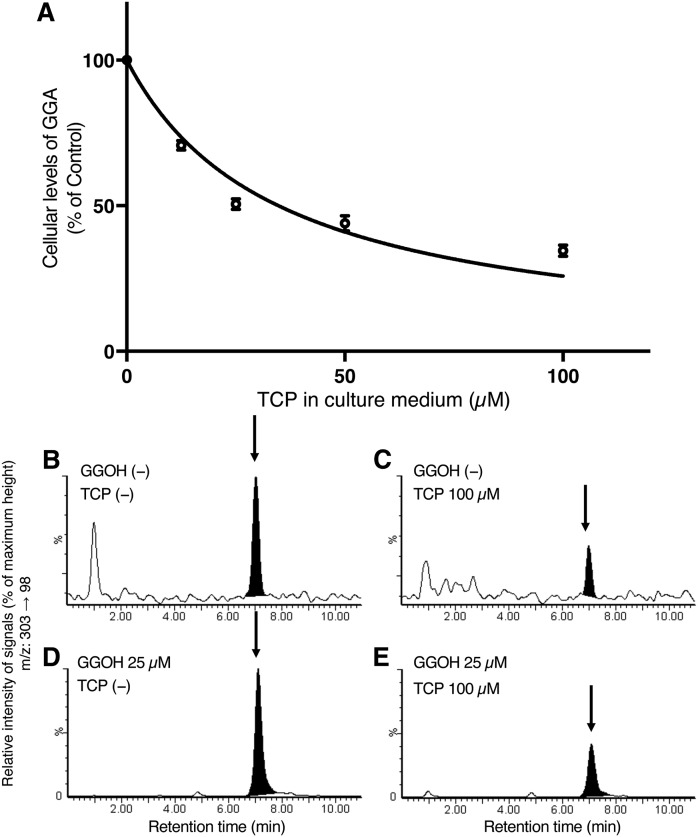
TCP-induced downregulation of cellular GGA levels in HuH-7 cells. A: Dose-dependent changes of endogenous GGA level in HuH-7 cells after treatment with 0–100 μM of TCP for 24 h. The amount of the intracellular GGA represents the mean ± SD of three measurements. The IC_50_ of TCP was obtained using GraphPad Prism 7.0. B–E: LC/MS/MS chromatograms of the lipid extracts from the nontreated control HuH-7 cells (B), cells treated for 48 h with 100 μM of TCP (C) with 25 μM GGOH for 24 h (D), and the cells treated for 24 h with 25 μM of GGOH after 24 h pretreatment with 100 μM of TCP (E). The arrows indicate the elution position of the authentic GGA. The vertical axes of the chromatograms show a signal intensity of 85 as 100% (B, C), and 2,210 as 100% (D, E).

First, we confirmed whether TCP, an inhibitor of MAOs, worked as a micromolar inhibitor of GGA biosynthesis in a cell culture system. **Figure 1A** clearly shows that TCP added in the culture medium decreased the cellular level of endogenous GGA in HuH-7 cells in a dose-dependent manner with an apparent IC_50_ of approximately 33 μM. Furthermore, 100 μM TCP not only decreased the endogenous GGA (Fig. 1B, C), but also suppressed the conversion of exogenously added GGOH to GGA in cultured HuH-7 cells (Fig. 1D, E).

### Downregulation of cellular GGA by *MAOB* siRNA

**Fig. 2.**
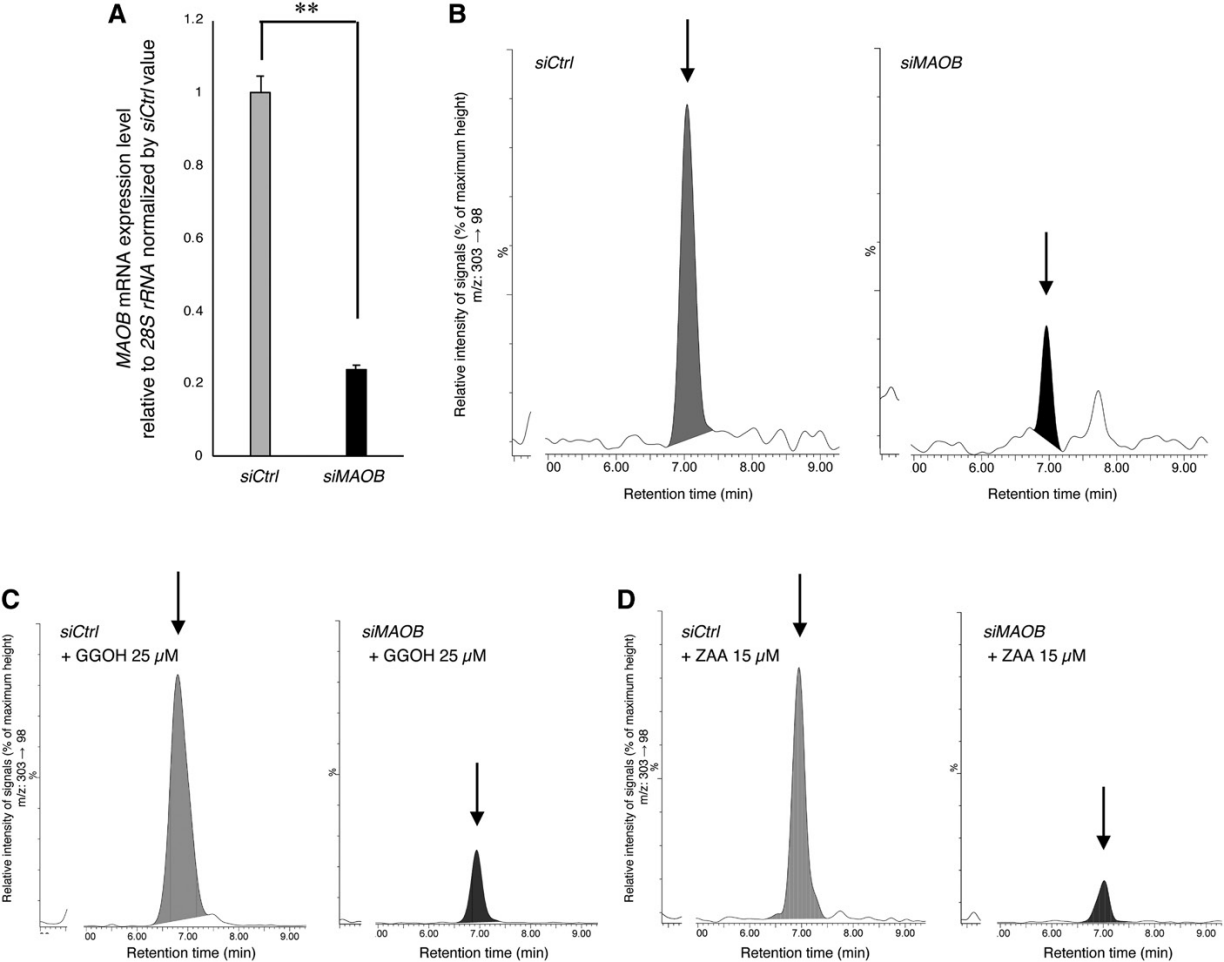
*MAOB* siRNA-induced downregulation of cellular GGA levels in HuH-7 cells. A: Total RNA was prepared from HuH-7 cells after 72 h incubation with each siRNA to measure the cellular levels of *MAOB* mRNA by QRTPCR. Each bar represents the mean ± SEM (n = 3). B–D: LC/MS/MS chromatograms of the lipid extract from each sample treated as described as follows. After HuH-7 cells were incubated with each siRNA for 96 h, the cells were incubated for another 24 h in the absence (B) or presence of 25 μM GGOH (C) or 15 μM ZAA (D). The arrows indicate the elution position of authentic GGA. The vertical axes of the chromatograms show a signal intensity of 60 as 100% (B), 1,460 as 100% (C), and 246 as 100% (D). **P < 0.01 compared with control (*siCtrl*).

Even though TCP decreased the cellular level of both endogenous GGA and exogenous GGOH-derived GGA in HuH-7 cells, we cannot exclude a possibility that some other TCP-sensitive enzymes, such as MAOA and cytochrome P450 (CYP, P450) enzymes, are involved in the biosynthesis of GGA. Therefore, we performed knockdown of the *MAOB* gene in HuH-7 cells using *MAOB* siRNA. The *MAOB* mRNA levels in HuH-7 cells transfected with *MAOB* siRNA were significantly decreased 72 h after the transfection (**Fig. 2A**), followed by a significant decrease in endogenous GGA at 120 h (*siCtrl* 8.95 ± 0.74 pmol/g; *siMAOB* 2.22 ± 0.18 pmol/g, *P* < 0.05; their representative LC/MS/MS chromatograms are shown in Fig. 2B). Furthermore, a dramatic increase in the intracellular GGA level induced by the exogenous GGOH was significantly suppressed by *MAOB* knockdown (*siCtrl* 205.14 ± 7.36 pmol/g; *siMAOB* 77.19 ± 3.36 pmol/g, *P* < 0.05; see Fig. 2C). In addition, *MAOB* knockdown significantly suppressed the squalene synthase inhibitor ZAA-induced accumulation of endogenous GGA (*siCtrl* 44.23 ± 1.36 pmol/g; *siMAOB* 13.37 ± 2.14 pmol/g, *P* < 0.05; the chromatograms are shown in Fig. 2D).

**Fig. 3.**
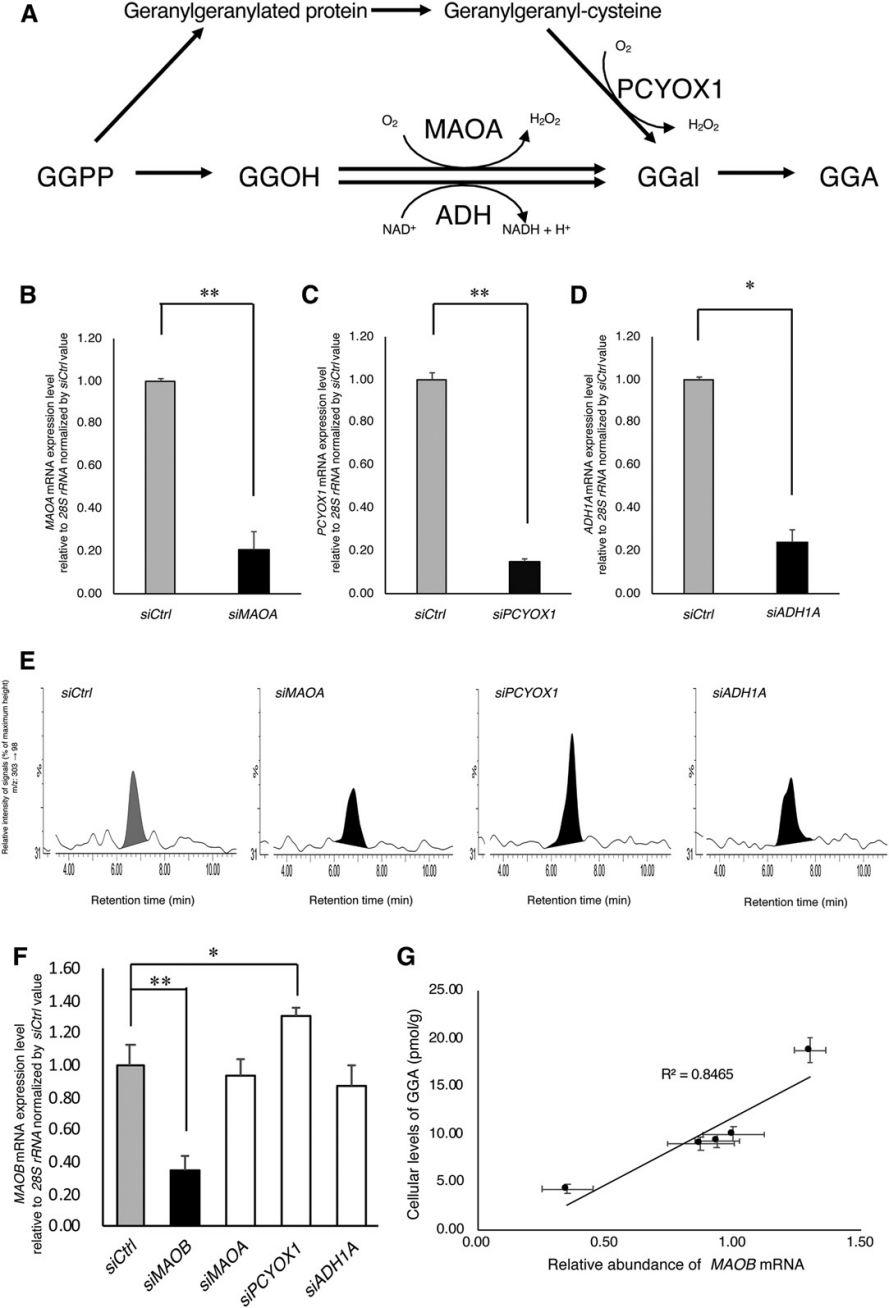
Knockdown of *MAOA*, *PCYOX1*, and *ADH1A* genes using each siRNA in HuH-7 cells does not induce a decrease in endogenous GGA level. A: According to the literature, possible metabolic pathways to produce GGal are depicted in addition to oxidation of GGOH to GGal catalyzed by MAOB. B–D: Total RNA was prepared after HuH-7 cells were incubated with each siRNA for 72 h to measure the cellular levels of *MAOA*, *PCYOX1*, and *ADH1A* mRNA by QRTPCR. Each bar represents the mean ± SEM (n = 3). E: LC/MS/MS chromatograms of the lipid extract from HuH-7 cells incubated with control siRNA (*siCtrl*), *MAOA* siRNA (*siMAOA*), *PCYOX1* siRNA (*siPCYOX1*), or *ADH1A* siRNA (*siADH1A*) for 120 h. The chromatograms show a peak height of 58 as 100% (*siCtrl*, *siMAOA*, *siADH1A*) and 84 as 100% (*siPCYOX1*). F: The expression level of *MAOB* mRNA in HuH-7 cells knocked down with each siRNA. Each bar represents the mean ± SEM (n = 3). G: Correlation between *MAOB* mRNA expression level and intracellular GGA level in HuH-7 cells knocked down with each siRNA. *P < 0.05 compared with control (*siCtrl*). **P < 0.01 compared with control (*siCtrl*) (*t*-test).

According to the literature (9, 11, 20, 21), MAOA, PCYOX1, and ADH1A are potential enzymes that generate GGal, a direct precursor of GGA (**Fig. 3A**). Hence, here, we knocked down each gene encoding these enzymes, and then measured the amount of endogenous GGA in each knockdown cell. Although the transfection of *MAOA* siRNA, *PCYOX1* siRNA, or *ADH1A* siRNA significantly reduced each gene mRNA level compared with negative control siRNA 72 h after transfection (Fig. 3B–D), the cellular levels of endogenous GGA in HuH-7 cells were not decreased in the *MAOA* siRNA-treated (9.23 ± 0.68 pmol/g) and *ADH1A* siRNA-treated (8.97 ± 0.72 pmol/g) cells and, unexpectedly, increased in the *PCYOX1* siRNA-treated (18.76 ± 2.32 pmol/g, *P* < 0.05) cells at 120 h (Fig. 3E), which is in stark contrast to the case of the *MAOB* knockdown (Fig. 2B). When the relative cellular level of *MAOB* mRNA in each siRNA-treated cell was examined, the *MAOB* expression level was significantly increased by *PCYOX1* siRNA treatment (Fig. 3F). When the intracellular levels of endogenous GGA were plotted against *MAOB* mRNA levels in these cells, a strong correlation was detected between endogenous GGA and *MAOB* mRNA levels (*r*^2^ = 0.8465; Fig. 3G).

### Catalytic activity of recombinant human MAOB in the oxidation of GGOH to GGal

**Fig. 4.**
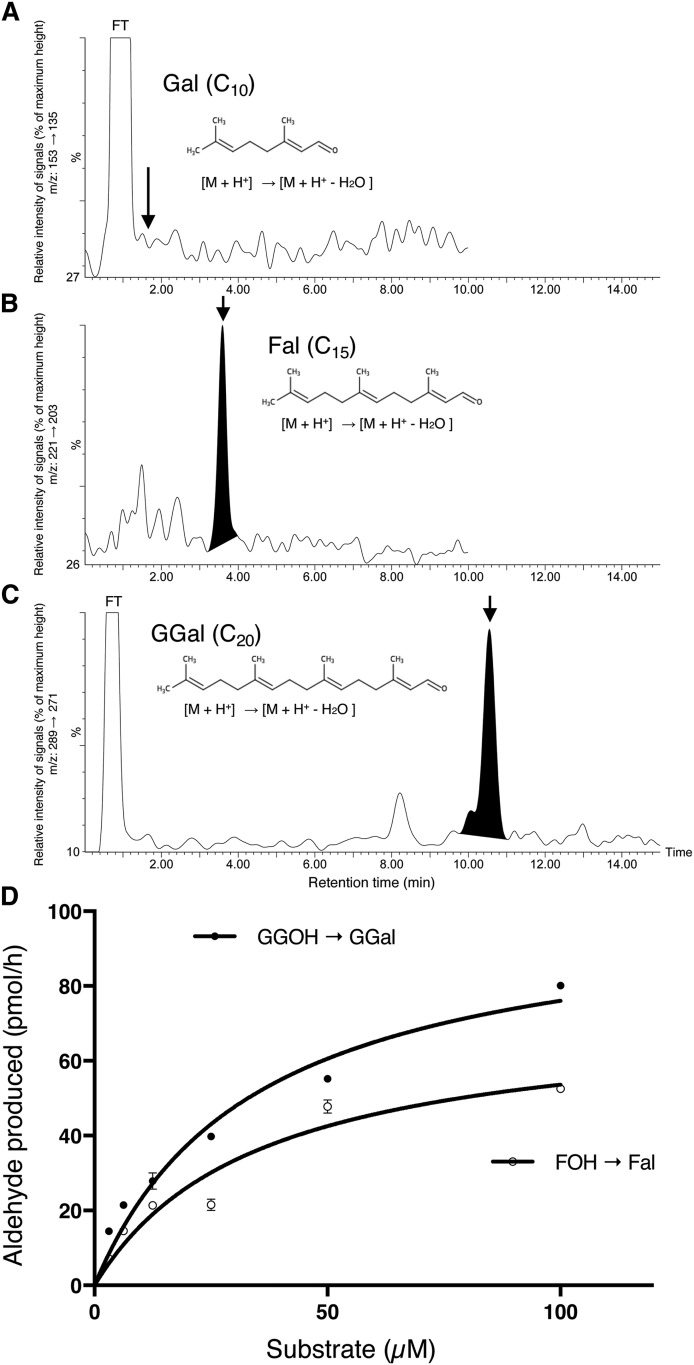
Recombinant human MAOB catalyzes oxidation of GGOH and FOH to GGal and Fal, respectively. LC/MS/MS elution profiles of ethanol extracts after incubation of the recombinant hMAOB with 25 μM GOH (A), 25 μM FOH (B), or 25 μM GGOH (C) at 37°C for 1 h. The elution position of each authentic aldehyde is indicated by arrows. D: The increasing concentrations of GGOH or FOH were incubated with 0.25 U hMAOB at 37°C for 1 h. The amounts of GGal or Fal products were measured by LC/MS/MS analysis. Values of *K_m_* were obtained by fitting plots of amounts of GGal or Fal products versus GGOH or FOH concentration, respectively, using GraphPad Prism 7. Each point represents the mean ± SD (n = 3). FT, flow through.

Because the results so far obtained in the present study strongly indicate that the MAOB enzyme is involved in GGA biosynthesis, we next decided to examine whether recombinant human MAOB protein is able to catalyze the oxidation reaction of acyclic isoprenols by increasing the isoprene unit from 2 to 4 to produce the corresponding aldehydes. As a result, MAOB had no ability to oxidize GOH (C_10_-acyclic monoterpenol; **Fig. 4A**), but undoubtedly produced Fal (Fig. 4B) from FOH (C_15_-acyclic sesquiterpenol) and GGal (Fig. 4C) from GGOH (C_20_-acyclic diterpenol). Then, kinetic analysis using increasing concentrations of GGOH or FOH as a substrate was performed. As shown in Fig. 4D, a reaction that oxidizes GGOH to GGal showed Michaelis-Menten-type kinetics (Fig. 4D) and the *K_m_* value of the recombinant human MAOB was calculated to be 34.34 ± 5.35 μM for GGOH, which is in the range of those of rat hepatic (10) and human hepatoma GGOH oxidase (11). The kinetic analysis also demonstrated that the *K_m_* value was 35.22 ± 7.77 μM for FOH, which is an established inhibitor that competitively inhibits MAOB activity (22–24). Although the same amount of the recombinant protein was used in the enzyme assay, *V_max_* was calculated to be 72.51 ± 6.87 pmol/h for FOH and 102.2 ± 6.77 pmol/h for GGOH (Fig. 4D), indicating that the turnover number should be 1.4-times greater for GGOH than for FOH.

### *MAOB* gene KO by the CRISPR/Cas9-HDR system and the resultant cellular endogenous GGA changes

**Fig. 5.**
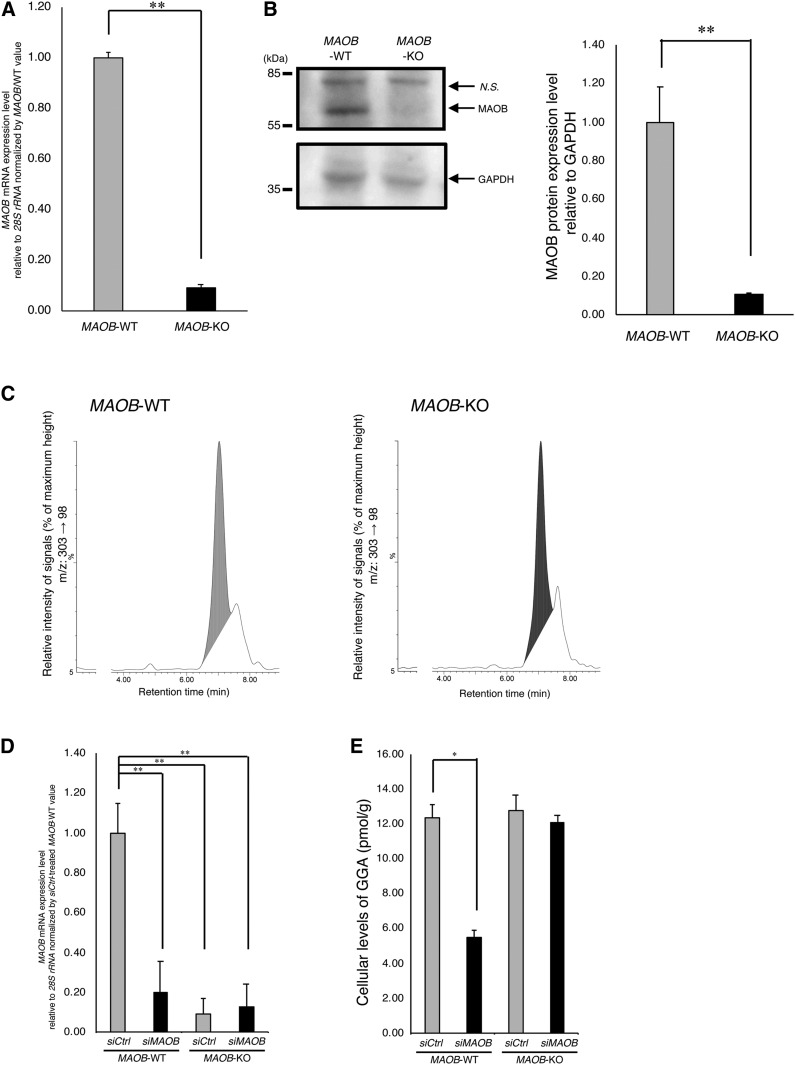
KO of *MAOB* by the CRISPR/Cas9-HDR system in Hep3B cells significantly reduces the cellular level of both *MAOB* mRNA and protein, but does not induce a decrease of intracellular GGA. A: *MAOB* mRNA expression level in *MAOB*-KO cells established by the CRISPR/Cas9-HDR system and WT Hep3B cells. Each bar represents the mean ± SEM (n = 3). B: Total RIPA cell lysates of Hep3B *MAOB*-WT cells and Hep3B *MAOB*-KO cells were analyzed by Western blotting to measure the level of MAOB. *N.S.* is a nonspecific band. Each bar in the right panel represents the mean ± SEM (n = 3) of the relative intensity of the MAOB band. C: LC/MS/MS chromatograms of the lipid extract from Hep3B/*MAOB*-WT and Hep3B/*MAOB*-KO. The vertical axes of both chromatograms show a signal intensity of 184 as 100%. D: The relative expression levels of *MAOB* mRNA to that of *siCtrl*-treated Hep3B/*MAOB*-WT cells upon *MAOB* siRNA treatment in Hep3B/*MAOB*-WT or Hep3B/*MAOB*-KO for 72 h. Each bar represents the mean ± SEM (n = 3). E: The endogenous GGA levels of the lipid extract from Hep3B/*MAOB*-WT or Hep3B/*MAOB*-KO after incubation with *MAOB* siRNA for 120 h. The amount of the intracellular GGA represents the mean ± SD of three measurements. *P < 0.05 compared with control (Hep3B/*MAOB*-WT *siCtrl*). **P < 0.01 compared with control (Hep3B/*MAOB*-WT *siCtrl*).

From the above results, we were convinced that MAOB is at least partly involved in GGA biosynthesis in HuH-7 cells. However, the above knockdown experiments just showed transient changes, so we next attempted to induce stable depletion of endogenous GGA in human hepatoma cells by establishing *MAOB*-KO cell clones using the CRISPR/Cas9-HDR system. Because *MAOB*-KO cells could not be established in HuH-7 cells by puromycin selection after several trials, another human hepatoma-derived cell line, Hep3B, which does not harbor any translocation in the X chromosome, was used because the *MAOB* gene is located on the X chromosome. A puromycin-resistant clone of Hep3B was successfully obtained and it dramatically reduced *MAOB* expression at both the mRNA and protein levels (**Fig. 5A**, B). However, unexpectedly, the endogenous GGA of the KO cells (Hep3B/*MAOB*-KO, 11.82 ± 0.84 pmol/g) did not decrease at all compared with the WT Hep3B cells (Hep3B/*MAOB*-WT, 12.51 ± 0.82 pmol/g) (Fig. 5C).

To exclude the possibility that a *MAOB* siRNA-induced reduction of the endogenous GGA level in HuH-7 cells was due to its off-target effect, we conducted further knockdown experiments using the *MAOB* siRNA in Hep3B/*MAOB*-KO and Hep3B/*MAOB*-WT. The *MAOB* siRNA induced a significant decrease in *MAOB* mRNA levels followed by a significant decrease in the intracellular GGA also in Hep3B/*MAOB*-WT (*siCtrl* 12.32 ± 0.77 pmol/g; *siMAOB* 5.50 ± 0.43 pmol/g, *P* < 0.05). However, the *MAOB* siRNA did not further suppress the cellular *MAOB* mRNA level that was hardly expressed in the KO cells (Fig. 5D), nor did it cause a decrease in endogenous GGA of Hep3B/*MAOB*-KO (*siCtrl* 12.78 ± 0.86 pmol/g; *siMAOB* 12.07.50 ± 0.44 pmol/g, Fig. 5E), suggesting that the *MAOB* siRNA-induced reduction of endogenous GGA was not due to its off-target effect in both HuH-7 and Hep3B cells.

Next, to investigate another possibility that a metabolic redundancy of GGA biosynthesis by other enzyme(s) was evoked in the Hep3B/*MAOB*-KO cells, we conducted further knockdown experiments using each siRNA of some potential enzymes (shown in Fig. 3A) that may produce GGal in Hep3B/*MAOB*-KO and Hep3B/*MAOB*-WT. As for other enzymes potentially involved in GGA biosynthesis, efficient knockdown of each gene did not cause any decrease in endogenous GGA either in Hep3B/*MAOB*-WT or Hep3B/*MAOB*-KO (see supplemental Fig. S2).

### Back-transfection of the *MAOB* gene into *MAOB*-KO cells restores the MAOB dependence of intracellular GGA

**Fig. 6.**
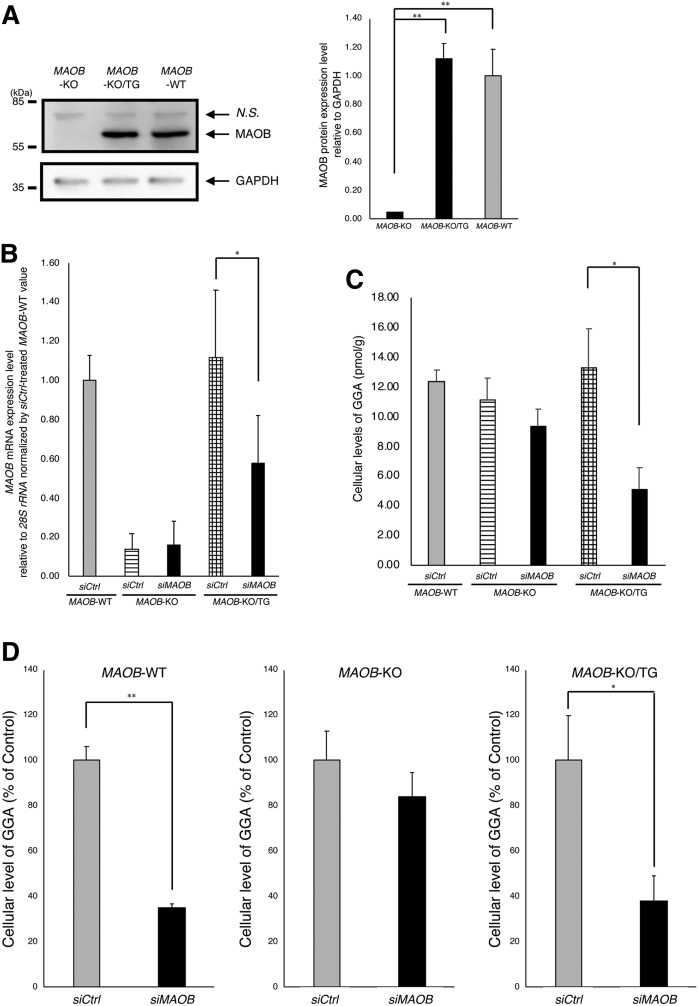
Rescue of *MAOB* siRNA sensitivity of the cellular GGA level in Hep3B/*MAOB*-KO cells by transgenic *MAOB* expression. A: Total RIPA cell lysates of Hep3B/*MAOB*-KO, Hep3B/*MAOB*-KO/TG, and Hep3B/*MAOB*-WT were analyzed by Western blotting with an anti-MAOB antibody. GAPDH was analyzed as a loading control. Each bar in the right panel represents the mean ± SEM (n = 3) of the relative intensity of the MAOB band to that of the corresponding GAPDH band. B: The relative expression levels of *MAOB* mRNA upon *MAOB* siRNA treatment in Hep3B/*MAOB*-WT, Hep3B/*MAOB*-KO, or Hep3B/*MAOB*-KO/TG. Each bar represents the mean ± SEM (n = 3). C: The endogenous GGA levels in the lipid extract from Hep3B/*MAOB*-WT, Hep3B/*MAOB*-KO, or Hep3B/*MAOB*-KO/TG incubated with *MAOB* siRNA or control siRNA for 120 h. Each bar represents the mean ± SEM (n = 3). D: Changes in intracellular GGA level by *MAOB* siRNA treatment in Hep3B/*MAOB*-WT, Hep3B/*MAOB*-KO, or Hep3B/*MAOB*-KO/TG. The amount of intracellular GGA is represented as percent of the control (the mean ± SD of three measurements). *P < 0.05 compared with control. **P < 0.01 compared with control (ANOVA with post hoc Scheffe).

Finally, to understand how tightly the *MAOB* gene is linked to metabolic maintenance of the intracellular level of endogenous GGA, Hep3B/*MAOB*-KO/TG cells were established by transfecting Hep3B/*MAOB*-KO with the *MAOB* gene expression plasmid. The back-transfection of the *MAOB* gene into the KO cells restored the MAOB protein level in the KO cells to the level of the WT cells (**Fig. 6A**), in accordance with the cellular *MAOB* mRNA levels (Fig. 6B). Knockdown of the transgenic *MAOB* gene in Hep3B/*MAOB*-KO/TG cells with the *MAOB* siRNA significantly reduced the cellular level of the *MAOB* mRNA level (Fig. 6B) as well as the endogenous GGA level (*siCtrl* 13.28 ± 2.61 pmol/g; *siMAOB* 5.06 ± 1.48 pmol/g, *P* < 0.05, Fig. 6C). Regarding the nature of WT cells (*MAOB*-WT), of which *MAOB* siRNA reduces intracellular GGA content, the KO cells (*MAOB*-KO) were rescued to the same extent as WT cells by back-transfection of the *MAOB* gene into the KO cells (*MAOB*-KO/TG), as clearly illustrated in Fig. 6D.

## DISCUSSION

In the present study, we provide concrete evidence that hepatic MAOB is involved in the biosynthesis of GGA, which is believed to prevent hepatocarcinogenesis. Suppression of MAOB activity by either MAO inhibitor TCP or *MAOB* siRNA significantly reduced endogenous GGA levels in human hepatoma cells. However, the amount of intracellular GGA was not reduced in *MAOB*-KO (Hep3B/*MAOB*-KO) cells compared with *MAOB*-WT (Hep3B/*MAOB*-WT) cells. Interestingly, back-transfection of the *MAOB* gene into Hep3B/*MAOB*-KO cells completely restored the *MAOB* siRNA-mediated reduction of endogenous GGA, strongly suggesting that the *MAOB* gene is the enzyme primarily responsible for maintenance of the endogenous GGA level in human hepatoma cells.

As described in the Introduction, we recently reported that, among rat organs, endogenous GGA is rich in the liver and, in human hepatoma-derived cells, its biosynthesis from MVA via FPP and GGPP was confirmed by isotopomer spectral analysis (7). However, at the start of our GGA research, GGA was chemically synthesized as a preventive drug for second primary hepatoma together with peretinoin or 4,5-didehydroGGA, which was indeed evaluated in several placebo-controlled randomized clinical trials with positive results (4, 5, 25, 26). Then, we reported the natural occurrence of GGA in several medicinal herbs (6). In 2019, we reported that endogenous GGA can be metabolically labeled from ^13^C-MVA via ^13^C-GGPP in the MVA pathway in mammalian cells (7). GGPP was reported to be directly converted to alcoholic GGOH by GGPPase in rat liver microsomes (8). Although GGOH was first reported to be oxidized by ADH (9), we have provided several lines of evidence for the possibility that MAOB may catalyze the oxidation of GGOH to GGal (10, 11). The reason we started thinking that MAOB might be involved in the oxidation of amine-free GGOH, a 20-carbon acyclic isoprenol, is that FOH, a 15-carbon acyclic isoprenol in tobacco smoke, has been identified as a selective inhibitor against MAOB (22–24). X-ray crystallography illustrates that FOH is inserted into the substrate-binding pocket of MAOB and is close to the cofactor FAD site, and these authors described that “if the bound *trans*,*trans*-FOH is considered as a substrate mimic, these structural data provide support for the polar nucleophilic mechanism” (22, p.15765). Hence, we hypothesized that GGOH is one isoprene unit longer than FOH, so that a distance between the 1-CH of GGOH and the N(5) of the flavin in the binding site of MAOB is shorter than the 3.4 Å, which is a distance between the 1-CH of the bound FOH and the N(5) of the flavin in FAD attached to MAOB. Such a topological speculation predicts the 1-CH of GGOH to be oxidized and converted to GGal by the proposed polar nucleophilic attack mechanism (22). We have previously reported that the recombinant hMAOB protein definitely oxidized GGOH to GGal and the activity was inhibited by TCP, an inhibitor of MAO, and the same was true with mitochondrial fractions of HuH-7 cells as enzyme sources (11).

In this study, we decided to show that MAOB enzyme activity is involved in the oxidation reaction from GGOH to GGal using cell culture systems. We first tested to determine whether TCP of the MAO inhibitor inhibits the GGA biosynthetic pathway in a cell-culture system. As described in the Results, TCP induced dose-dependent downregulation of the endogenous GGA content in HuH-7 cells with an IC_50_ of 33 μM (Fig. 1A). However, the cellular GGA was not completely depleted by TCP treatment even at 100 μM (Fig. 1B, C), and at over 50 μM, the changes in the cellular GGA amount became marginal. Hence, we supposed that some part (roughly 40%) of endogenous GGA was produced by enzymes other than MAOB. After subtraction of this value, the corrected IC_50_ of TCP was recalculated to be 10.7 μM, which is in the range of the IC_50_ of TCP for MAOB enzyme activity (7.0 μM) (27). It is interesting that a similar proportion of the cellular GGA from exogenous GGOH remained after the 100 μM TCP treatment (Fig. 1D, E). These results indicate that TCP-sensitive enzymes are partly involved in the oxidation of GGOH to GGal to produce the cellular GGA, but we cannot yet conclude that the TCP-sensitive enzyme is MAOB. Because, in addition to MAOB, TCP-sensitive enzymes include MAOA (IC_50_ = 11.5 μM) (27), CYP2A6 (IC_50_ = 0.42 μM) (28), and CYP2E1 (IC_50_ = 3.0 μM) (28).

The next knockdown experiment provided additional strong evidence that the *MAOB* gene is mainly responsible for maintenance of the cellular GGA level in HuH-7 cells. In other words, *MAOB* siRNA-mediated downregulation caused more than an 80% reduction of not only the endogenous GGA level but also the intracellular GGA levels upregulated by either ZAA or exogenous GGOH (Fig. 2), suggesting that the greater part of the cellular GGA was produced through a MAOB-mediated process. Nevertheless, we still saw that a certain amount of GGA remained in the cells after the downregulation of the *MAOB* gene.

In the literature (9, 11, 20, 21), several enzymes other than MAOB are reported to be able to produce GGal (Fig. 3A); however, knockdown of these genes did not change the intracellular GGA (Fig. 3B–E), except that knockdown of the *PCYOX1* gene significantly and inversely upregulated the GGA level. Although we were able to show how the downregulation of *PCYOX1* gene expression is linked to the upregulation of the cellular GGA level, at present we cannot determine how the downregulation of the *PCYOX1* gene resulted in the upregulation of *MAOB* gene expression (Fig. 3F). Regardless, the cellular levels of endogenous GGA are significantly correlated with the expression levels of the *MAOB* gene in any knocked down cells (Fig. 3G).

We are now quite confident that MAOB is a putative enzyme responsible for biosynthesis of GGA in human hepatoma cells. Using LC/MS/MS, we confirmed our previous findings that recombinant human MAOB is active in oxidizing GGOH to GGal (11). In the present study, we provide additional evidence that MAOB catalyzes the oxidation reaction of acyclic isoprenol by showing that FOH is an additional substrate in addition to GGOH, although GOH was not oxidized to Gal. Considering that recombinant human MAOA did not oxidize GGOH (11) and the catalytic efficiency (*kcat*/*K*m) of the recombinant hMAOB enzyme was calculated to be approximately 1.7 times greater for GGOH than for geranylgeranylamine (unpublished results), we suggest that the recombinant hMAOB recognizes FOH and GGOH as a specific substrate and catalyzes the oxidation reaction. As for other acyclic isoprenols longer than GGOH, we have not tried to use them as substrates for MAOB.

As a third step, we knocked out the *MAOB* gene in the hepatoma cell and attempted to deplete the intracellular GGA, but we failed to deplete it using the KO method. The *MAOB*-KO Hep3B (Hep3B/*MAOB*-KO) cells established by the CRISPR/Cas9-HDR system, drastically reduced both the mRNA and protein levels of the *MAOB* gene, as expected. However, the amount of endogenous GGA content in Hep3B/*MAOB*-KO cells was unexpectedly not reduced, which is apparently inconsistent with the *MAOB*-knockdown-mediated reduction of the endogenous GGA level in HuH-7 cells. Thus, we considered and tested three possibilities: *1*) Hep3B cells may produce endogenous GGA using enzyme(s) other than MAOB, which is different from HuH-7 cells. *2*) The decrease in the endogenous GGA content in HuH-7 cells by *MAOB* siRNA may be due to an off-target effect. *3*) When the *MAOB* gene is knocked out, other GGal-producing enzymes may be upregulated to compensate for the absence of MAOB activity, and this putative compensatory mechanism may help maintain the original GGA level in Hep3B/*MAOB*-KO cells.

Among the three possibilities described above, the first was immediately excluded because introducing *MAOB* siRNA significantly reduced the intracellular GGA level in Hep3B/*MAOB*-WT to the same extent as in HuH-7 cells. However, *MAOB*-siRNA-treated Hep3B/*MAOB*-KO cells did not change the amount of intracellular GGA, implying that there are no off-targets other than the *MAOB* gene of the *MAOB* siRNA used in the present study to refute the second possibility. Finally, to verify the third possibility, we knocked down the other GGal-producing *ADH1A* and *PCYOX1* genes or the *MAOA* gene in Hep3B/*MAOB*-KO cells, but we found no change in the cellular GGA content of the KO cells (see supplemental Fig. S2). At present, a compensatory mechanism for maintaining the GGA concentration in the KO cells has not been proved, but enzymes of the CYP family are under investigation on the premise that a compensatory mechanism does exist.

In this context, the questions arose as to whether MAOB was no longer needed for GGA biosynthesis once putative compensatory mechanisms began to work, and whether MAOB or a compensatory mechanism(s) is dominant in GGA biosynthesis. To answer these questions, we performed back-transfection of the *MAOB* gene into Hep3B/*MAOB*-KO cells to make Hep3B/*MAOB*-KO/TG cells. As a result, although Hep3B/*MAOB*-KO/TG cells showed no change in the intracellular GGA content compared with either Hep3B WT cells or *MAOB*-KO cells, the *MAOB* siRNA-mediated knockdown of the transgenic *MAOB* significantly reduced intracellular GGA levels in Hep3B/*MAOB*-KO/TG. Thus, the back-transfection of the *MAOB* gene completely rescued the KO cells from *MAOB* siRNA insensitivities of endogenous GGA (Fig. 6D). From these results, we conclude that a MAOB-mediated metabolic pathway is a primary process for maintaining the cellular GGA level in human hepatoma cells. Although it was not possible to identify a putative compensatory enzyme in this study, it is absolutely essential to prove it in the future to establish GGA biosynthesis. Whatever compensatory enzyme works in the *MAOB*-KO cells, the existence of a putative maintenance mechanism at the cellular level of endogenous GGA in the *MAOB*-KO cells suggests that GGA is an essential metabolite that has a vital function for cell life other than cell death induction in malignant cells. Indeed, we recently published our observations about another biological function of GGA apart from its function in malignant cells; that is, dietary supplementation with GGA during mating, pregnancy, and lactating periods significantly improved the reproduction index in C3H/HeN mice (29) and SAM P1 mice (30).

Finally, let us consider why the CRISPR/Cas9-HDR method worked in the Hep3B cell line, but failed to knock out the *MAOB* gene in the HuH-7 cell line. As for the gene encoding MAOB, of note, there is an important difference between HuH-7 and Hep3B cell lines. The *MAOB* gene localizes to a short arm (Xp11.3) of the X-chromosome and a Hep3B cell harbors a single intact X-chromosome (31, 32), whereas in HuH-7 cells, many derivative chromosomes involving X-chromosome [der(X)t(X:14), +der(X)t(X;19), +der(4)t(X;4), der(11)t(X;11), der(13)t(X;13)] are reported (33). These derivative chromosomes may make it difficult for the CRISPR/Cas9-HDR method to knock out the *MAOB* gene in HuH-7 cells. Therefore, we think a molecular mechanism underlying oxidation of GGOH may be the same between these two cell lines and the same enzyme system may exist even in normal hepatocytes.

In conclusion, herein, we show that MAOB is principally involved in GGA biosynthesis through oxidation of GGOH in human hepatoma cells. Historically, it has been established that MAOB is involved in the degradation of catecholamines in the brain (34), and in this context, the hepatic-MAOB function is speculated to work in the degradation of amine-containing xenobiotics in bulk, despite its highest expression level. The present study clearly demonstrates that hepatic MAOB has a completely new metabolic role that has not been reported so far. This role is in the biosynthesis of GGA, a biologically active isoprenoid lipid. Therefore, we propose with confidence that hepatic MAOB is also a GGOH oxidase.

## Supplementary Material

Supplemental Data
