# Supplementary material for: Hepatic monoamine oxidase B is involved in endogenous geranylgeranoic acid synthesis in mammalian liver cells
Source: J Lipid Res. 2020 Feb 24;61(5):778–89. doi: 10.1194/jlr.RA119000610 (PMC7193968; doi:10.1194/jlr.RA119000610)
Supplement: Supplemental Data [file supp_61_5_778__index.html]

Hepatic monoamine oxidase B is involved in endogenous geranylgeranoic acid synthesis in mammalian liver cells — Hepatic MAOB is involved in biosynthesis of GGA — Hepatic monoamine oxidase B is involved in endogenous geranylgeranoic acid synthesis in mammalian liver cells — Supplemental Data 

# Hepatic monoamine oxidase B is involved in endogenous geranylgeranoic acid synthesis in mammalian liver cells

## Supplemental Data

- Supplemental Table S1 - The sequences of each siRNA used for knockdown experiments.
- Supplemental Tables S2-S4 - Table. S2. The nucleotide sequences of each primer used for real-time RT-qPCR. Table. S3. The condition of thermal cycler for real-time RT-PCR of MAOA, MAOB, PCYOX1, and ADH1A. Table. S4. The condition of thermal cycler for real-time RT-PCR of 28S rRNA.
- Supplemental Table S5 - MRM mode conditions, and transition for each compound.
- Supplemental Figure S1 - Images of fluorescent microscopy for Hep3B cells. (A)Hep3B/MAOB-WT. (B)Hep3B/MAOB-KO expressing RFP. HDR plasmid is tagged with RFP.
- Supplemental Figure S2 - Knockdown of MAOA, PCYOX1, and ADH1A using each corresponding siRNA in Hep3B MAOB-KO cells does not induce a decrease in endogenous GGA. The relative expression level of each target mRNA upon A, MAOA siRNA, C, PCYOX1 siRNA and E, ADH1A siRNA treatment in Hep3B MAOB-WT cells or Hep3B MAOB-KO cells. Each bar represents the mean &#x00B1; SEM (n = 3). The endogenous GGA level of the lipid extract from Hep3B MAOB-WT cells or Hep3B MAOB-KO cells incubated with B, MAOA siRNA, D, PCYOX1 siRNA and F, ADH1A siRNA for 120 h. The amount of intracellular GGA represents the mean &#x00B1; SD of three measurements. \*, p &lt; 0.05 compared with control (siCtrl). \*\*, p &lt; 0.01 compared with control (siCtrl) (ANOVA with post hoc Scheffe).
