# Supplementary material for: Hepatic monoamine oxidase B is involved in endogenous geranylgeranoic acid synthesis in mammalian liver cells
Source: J Lipid Res. 2020 Feb 24;61(5):778–89. doi: 10.1194/jlr.RA119000610 (PMC7193968; doi:10.1194/jlr.RA119000610)
Supplement: Supplemental Data [file supp_RA119000610_158034_2_supp_476282_q5qfrh.docx]

Table. S1. The sequences of each siRNA used for knockdown experiments.

|  |  | Sequence (5’ – 3’) |
| --- | --- | --- |
| *siMAOA* | Sense A | GUGACAACAUCAUCAUAGATT |
|  | Antisense A | UCUAUGAUGAUGUUGUCACTT |
|  | Sense B | CGACUUCUCUAGACAUCUATT |
|  | Antisense B | UAGAUGUCUAGAGAAGUCGTT |
|  | Sense C | GGAUCCUUGUCAGUUGUAATT |
|  | Antisense C | UUACAACUGACAAGGAUCCTT |
| *siMAOB* | Sense A | GCAUGAAGAUUCACUUCAATT |
|  | Antisense A | UUGAAGUGAAUCUUCAUGCTT |
|  | Sense B | CUCUGCCAAUGAUGAGAAATT |
|  | Antisense B | UUUCUCAUCAUUGGCAGAGTT |
|  | Sense C | GGCUUAGCGUUCUGUUUCATT |
|  | Antisense C | UGAAACAGAACGCUAAGCCTT |
| *siPCYOX1* | Sense A | GGAAAGAUGUGAAGAUAGATT |
|  | Antisense A | UCUAUCUUCACAUCUUUCCTT |
|  | Sense B | CCACUCCGUUGAAUCGAAATT |
|  | Antisense B | UUUCGAUUCAACGGAGUGGTT |
|  | Sense C | GAAGCCCAAUCUGUAUCAATT |
|  | Antisense C | UUGAUACAGAUUGGGCUUCTT |
| *siADH1A* | Sense A | GCCUCUAGAGAAAGUCUGUTT |
|  | Antisense A | ACAGACUUUCUCUAGAGGCTT |
|  | Sense B | CCGUACCAUUCUGAUGUUUTT |
|  | Antisense B | AAACAUCAGAAUGGUACGGTT |
|  | Sense C | GCAACAGCUGGGAAAUAUCTT |
|  | Antisense C | GAUAUUUCCCAGCUGUUGCTT |
