# Supplementary material for: Hepatic monoamine oxidase B is involved in endogenous geranylgeranoic acid synthesis in mammalian liver cells
Source: J Lipid Res. 2020 Feb 24;61(5):778–89. doi: 10.1194/jlr.RA119000610 (PMC7193968; doi:10.1194/jlr.RA119000610)
Supplement: Supplemental Data [file supp_RA119000610_158034_2_supp_476283_q5qfrh.docx]

Table. S2. The nucleotide sequences of each primer used for real-time RT-qPCR.

| Genes | Primer | Sequence (5’ – 3’) |
| --- | --- | --- |
| *MAOA* | F | CAGCTCCTGGTGGAGAGACTA |
|  | R | TGGGTTGGTCCCACATAAGC |
| *MAOB* | F | GCAACAAATGCGACGTGGT |
|  | R | AGGATCCTCCAAGGTCCACA |
| *PCYOX1* | F | TGAGGAGAGCAACTGGTTCA |
|  | R | ACTGGTAGCGGTAGATCCTCA |
| *ADH1A* | F | TCTGGGAAAAGTATCCGTACCATT |
|  | R | TGAAGACTGCCACAAGGGAA |
| *28S rRNA* | F | TTAGTGACGCGCATGAATGG |
|  | R | TGTGGTTTCGCTGGATAGTAGGT |

F: forward primer, R: reverse primer

Table. S3. The condition of thermal cycler for real-time RT-PCR of *MAOA*, *MAOB*, *PCYOX1*, and *ADH1A*.

|  | Temperature, Duration | Slope |
| --- | --- | --- |
| Denature | 95ºC, 600 s | 20ºC / s |
| PCR (40 cycles) | 95ºC, 15 s | 20ºC / s |
|  | 60ºC, 60 s | 20ºC / s |
| Melting | 95ºC, 0 s | 20ºC / s |
|  | 57ºC, 15 s | 20ºC / s |
|  | 98ºC, 0 s | - |
| Cooling | 40ºC, 30 s | 20ºC / s |

Table. S4. The condition of thermal cycler for real-time RT-PCR of *28S rRNA*.

|  | Temperature, Duration | Slope |
| --- | --- | --- |
| Denature | 95ºC, 600 s | 4.4ºC / s |
| PCR (40 cycles) | 95ºC, 10 s | 4.4ºC / s |
|  | 60ºC, 10 s | 2.2ºC / s |
|  | 72ºC, 3 s | 4.4ºC / s |
| Melting | 95ºC, 1 s | 4.4ºC / s |
|  | 65ºC, 15 s | 2.2ºC / s |
|  | 95ºC, 1 s | - |
| Cooling | 40ºC, 30 s | 4.4ºC / s |
