# Supplementary material for: Hepatic monoamine oxidase B is involved in endogenous geranylgeranoic acid synthesis in mammalian liver cells
Source: J Lipid Res. 2020 Feb 24;61(5):778–89. doi: 10.1194/jlr.RA119000610 (PMC7193968; doi:10.1194/jlr.RA119000610)
Supplement: Supplemental Data [file supp_RA119000610_158034_2_supp_476284_q5qfrh.docx]

Table. S5. MRM mode conditions, and transition for each compound.

| Compound | m/z | | | Charge | Cone voltage |  | Collision energy |
| --- | --- | --- | --- | --- | --- | --- | --- |
|  | Molecular ion | → | Fragment ion |  | (V) |  | (eV) |
| Geranylgeranoic acid | 303.11 | → | 98.11 | – | 36 |  | 12 |
| Geranylgeranial | 289.4 | → | 271.3 | + | 20 |  | 10 |
| Farnesal | 221.3 | → | 203.3 | + | 20 |  | 11 |
| Geranial | 153.2 | → | 135.2 | + | 20 |  | 10 |
