# Supplementary material for: Hepatic monoamine oxidase B is involved in endogenous geranylgeranoic acid synthesis in mammalian liver cells
Source: J Lipid Res. 2020 Feb 24;61(5):778–89. doi: 10.1194/jlr.RA119000610 (PMC7193968; doi:10.1194/jlr.RA119000610)
Supplement: Supplemental Data [file supp_RA119000610_158034_2_supp_476285_q5qfrh.docx]

A

B

RFP

RFP

DIC

DIC

MERGE

MERGE


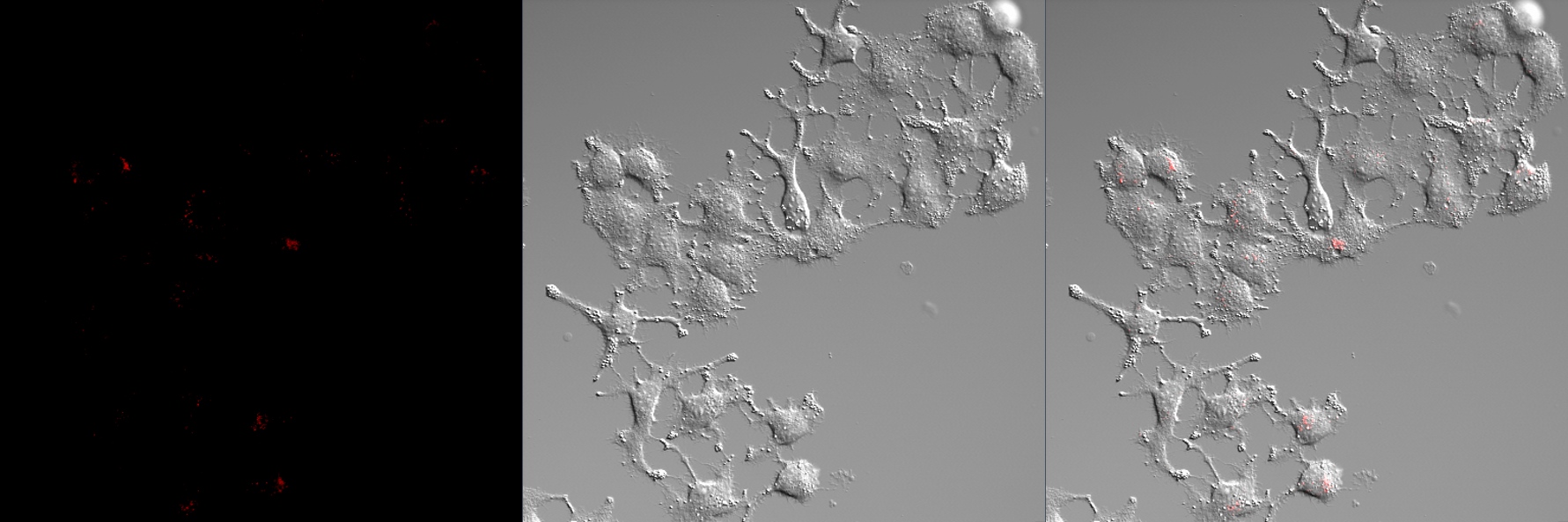


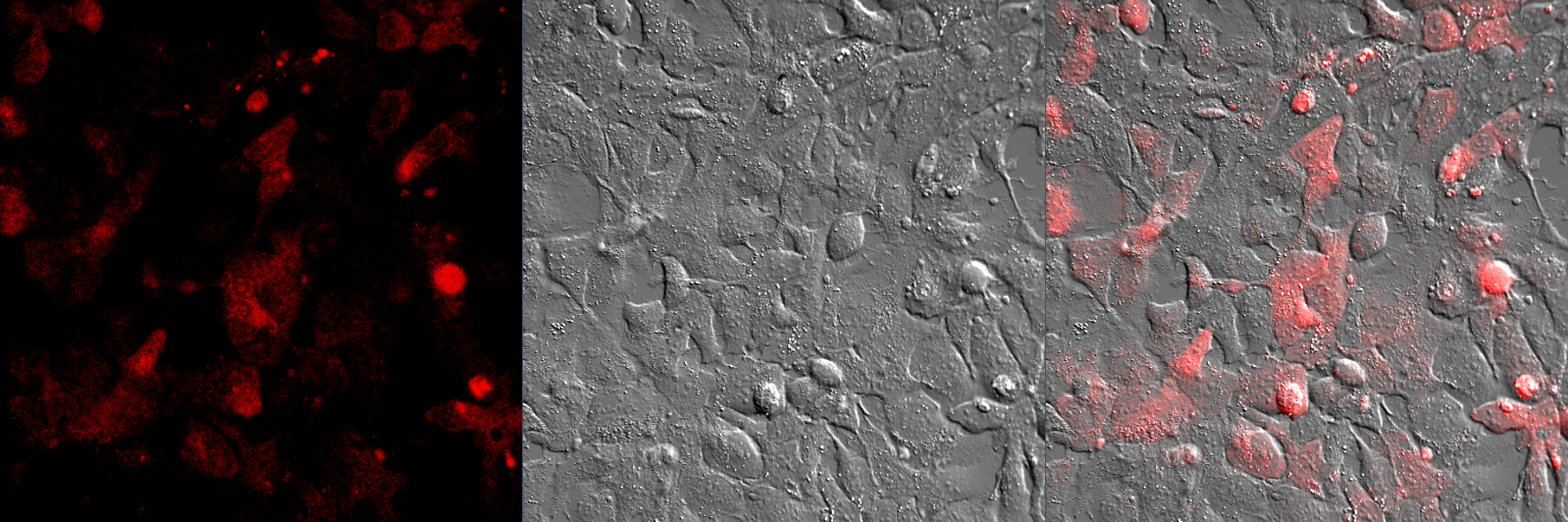


**Figure S1. Images of fluorescent microscopy for Hep3B cells.**

(*A*)Hep3B/MAOB-WT. (*B*)Hep3B/MAOB-KO expressing RFP. HDR plasmid is tagged with RFP.
