# Supplementary material for: Hepatic monoamine oxidase B is involved in endogenous geranylgeranoic acid synthesis in mammalian liver cells
Source: J Lipid Res. 2020 Feb 24;61(5):778–89. doi: 10.1194/jlr.RA119000610 (PMC7193968; doi:10.1194/jlr.RA119000610)
Supplement: Supplemental Data [file supp_RA119000610_158034_2_supp_476286_q5qfrh.docx]

**Figure S2. Knockdown of *MAOA*, *PCYOX1*, and *ADH1A* using each corresponding siRNA in Hep3B *MAOB*-KO cells does not induce a decrease in endogenous GGA.** The relative expression level of each target mRNA upon *A,* *MAOA* siRNA, *C,* *PCYOX1* siRNA and *E,* *ADH1A* siRNA treatment in Hep3B *MAOB*-WT cells or Hep3B *MAOB*-KO cells. Each bar represents the mean ± SEM (n = 3). The endogenous GGA level of the lipid extract from Hep3B *MAOB*-WT cells or Hep3B *MAOB*-KO cells incubated with *B,* *MAOA* siRNA, D*,* *PCYOX1* siRNA and *F,* *ADH1A* siRNA for 120 h. The amount of intracellular GGA represents the mean ± SD of three measurements. *, *p* < 0.05 compared with control (siCtrl). **, *p* < 0.01 compared with control (siCtrl) (ANOVA with post hoc Scheffe).
